# Changes in patient admissions after the 2015 Earthquake: a tertiary hospital-based study in Kathmandu, Nepal

**DOI:** 10.1038/s41598-020-61901-7

**Published:** 2020-03-18

**Authors:** Maria Moitinho de Almeida, Benjamin-Samuel Schlüter, Joris Adriaan Frank van Loenhout, Sunil Singh Thapa, K. C. Kumar, Ravikant Singh, Debarati Guha-Sapir, Deepak Prakash Mahara

**Affiliations:** 1 Centre for Research on the Epidemiology of Disasters, Institute of Health and Society, University of Louvain. 30, clos chapelle-aux-champs, 1200 Brussels, Belgium; 2 Center for Demographic Research (DEMO), University of Louvain, Louvain-la-Neuve, 1348 Belgium; 3 Department of Orthopedics, Tribhuvan University Teaching Hospital. Maharajgunj Rd, Kathmandu, 44600 Nepal; 4 Tribhuvan University Teaching Hospital. Maharajgunj Rd, Kathmandu, 44600 Nepal; 5 Doctors for You India, Natwar Parikh Compound, Near India Oil Nagar, Govandi. Mumbai, 400043 Maharashtra, India

**Keywords:** Health services, Public health

## Abstract

Literature on earthquake impact on hospital admissions is lacking, particularly in low-resource settings. Our aim was to study the pattern of admissions before and after the 2015 earthquake in a tertiary hospital in Nepal. We used routine hospital data from 9,596 admissions, and defined four periods: pre-earthquake (pre-EQ), acute (EQ1), post-acute (EQ2), and post-earthquake (post-EQ). We compared length of hospital stay (LOS) across the study periods using negative binomial regressions. We used logistic regressions to study changes in probability of admission for diagnostic categories, and Generalized Additive Models to model the difference in number of admissions compared to pre-EQ baseline. LOS was longer in EQ1 than during pre-EQ, in particular for injury-related admissions. In EQ1, the odds of injury admissions increased, while they decreased for the majority of other diagnoses, with the odds of pregnancy-related admissions remaining low until post-EQ. The number of admissions dropped in EQ1 and EQ2, and returned to pre-EQ trends in post-EQ, accumulating 381 admissions lost (CI: 206–556). Our findings suggest that hospital disaster plans must not only foresee injury management after earthquakes, but also ensure accessibility, in particular for pregnant women, and promote a quick return to normality to prevent additional negative health outcomes.

## Introduction

Violent, sudden-onset disasters, such as hurricanes or earthquakes, cause considerable damage in communities, leading to widespread destruction^1^. While deaths and direct injuries are immediate and expectable consequences of such disasters, drastic changes in the surrounding environment may have longer-term health effects^2–7^. These disasters also heavily disturb health systems, which must provide healthcare in a context of sudden increase of health needs, radical change of priority conditions, infrastructural and material damage, and staff shortages^8^. While most of the victims in need of care during and after a disaster are managed in an outpatient basis at emergency departments, a considerable amount of people will require hospitalization^9^. However, there is inconsistent evidence on the impact on hospital admissions immediately after and in the months following violent, sudden-onset disasters^3,10–12^.

Earthquakes are the most destructive type of natural disaster, having killed nearly 720,000 people globally between 2000 and 2018. About two thirds of all events worldwide occurred in Asia, where earthquakes affect much more people than earthquakes in all the other continents^13^. Rapid population growth, urbanization, poverty, and geological risks contribute to Asia’s seismic vulnerability.

Nepal is located on the boundaries of two colliding tectonic plates. On April 25^th^ 2015, an earthquake with a moment magnitude of 7.8 severely hit the country. The epicenter was in Gorkha district, and heavy infrastructural damage occurred in neighbouring regions, including in the capital city of Kathmandu located 76 km away. There were several aftershocks, the strongest occurring on May 12^th^. National health services were the only services available to treat the high number of injured victims in the first days, since external assistance arrived with a considerable delay^14^. The Tribhuvan University Teaching Hospital (TUTH) is a major tertiary hospital in Nepal, built with earthquake resistance standards, and was functioning immediately after the earthquake, having assisted many earthquake victims^15^. TUTH belonged to the Hospital Preparedness for Emergencies (HOPE) network since 2014^16^, and activated its disaster management plan after the earthquake, reorganizing its services and implementing a mass casualty triage system to categorize earthquake victims.

The literature portraying how earthquakes affect health service outputs, such as hospital admissions, is scarce in resource-poor settings. The available studies in Nepal either fail to show the longer term consequences of the earthquake on hospital admissions^17^, or do not capture the specificities of the initial days after the disaster^18,19^. Understanding the pattern of hospitalizations after earthquakes is essential to improve hospital preparedness and surge capacity in future disasters, and facilitates a quick return to regular activities. We studied the pattern of hospital admissions at TUTH from six weeks before to four months after the 2015 earthquake, in terms of length of hospital stay (LOS), diagnostic categories, and number of daily admissions.

## Methods

Data on hospital admissions between March 15^th^ and August 17^th^ 2015 were collected from centralized hospital registries. Three datasets were available: hospital admissions, hospital discharges, and admitted earthquake victims. The first dataset contained sociodemographic information and served as panel data for daily admissions. We linked it with the remaining datasets to include date of discharge and diagnosis. The victim dataset underwent hand verification in original patient files in case of missing data or inconsistent information. We identified common admissions and merged datasets through deterministic linkages of identification number, name, sex, age, and date of admission. If these did coincide, we considered them the same admission. If not, we only considered the entry from the hospital admission dataset.

Available variables were sex, age (continuous), date of admission, date of discharge, and diagnosis. In the discharge dataset, diagnosis was classified according to the International Classification of Diseases, 10^th^ revision (ICD-10), until the third level (letter and two digits), and was entered directly by TUTH’s administrative staff. In the earthquake victim dataset, diagnosis was originally entered as free text, and two independent researchers later codified it into ICD-10. If their coding differed, they would discuss and agree on a final code.

We classified age into four groups (0–4, 5–14, 15–49 and ≥50 years old), and used the 21 ICD-10 disease categories for our analyses. We calculated length of stay (LOS) as the number of days between hospital admission and hospital discharge. We defined the following four study periods:

1. Pre-earthquake (pre-EQ): admissions from March 15^th^ until April 24^th^ 2015
2. Acute earthquake period (EQ1): admissions between April 25^th^ (including) until May 15^th^ 2015
3. Post-acute earthquake period (EQ2): admissions from May 16^th^ (including) until June 5^th^ 2015
4. Post-earthquake (post-EQ): admissions from June 6^th^ until August 17^th^ 2015

We determined a six-week critical period divided into two three-week sub-periods for multiple reasons. First, six weeks after the earthquake, national health actors considered the critical period to be over, and health services started shifting back to a regular system^14^. It was also the time period required for other public services, such as schools, to reopen^20^. But most earthquake-related hospital admissions occur in the first days following the disaster^2^, and there were repeated aftershocks, the strongest on May 12^th^. Hence, we divided the critical six-week period in two smaller time intervals to increase precision and capture these particularities.

### Data analysis

First, we made a descriptive overview of the variables and tested for bivariate associations between sex, age groups, earthquake periods, and ICD-10 category, using chi-square tests.

#### Length of stay (LOS)

Length of hospital stay generally follows a right-skewed distribution curve. Negative binomial regression is suitable to analyze LOS, with the advantage over Poisson regression in case of overdispersion of counts^21^, which was the case in our sample: the variance-to-mean ratio ranged from 9.77 in pre-EQ to 15.86 in EQ1. We compared differences in Length of Stay in the four study periods using a Negative Binomial regression, and adjusted for age group, sex, and ICD-10 category. We also calculated LOS differences across the four periods for individual ICD-10 categories consisting of more than 10% of total admissions, adjusting for age group and sex. Outliers, i.e. admissions with a LOS longer than six months, were removed from the analysis (n = 4).

#### Association between EQ period and ICD-10 category

To evaluate how the probability of admission for a given ICD-10 category changed with earthquake period, in relation to all other ICD-10 categories, we carried out binary logistic regressions, adjusting for age group and gender. We assumed the outcome as a Bernoulli variable, where being admitted for a given ICD-10 category was a success and for any other ICD-10, a failure. We only included ICD-10 categories that followed the one in ten rule, in this case with a minimum of 70 observations, and each time we compared one category with all the others grouped. We tested for interaction terms between control variables.

#### Earthquake impact on hospital admissions

We calculated the median number of daily admissions for weekdays and Saturdays in the pre-EQ period, since Saturday is the only non-working day in Nepal. We considered these values as the baseline number of daily admissions. We computed the difference between this baseline and the actual number of daily admissions during the three following periods, and used Generalized Additive Models (GAM) to model these differences and to capture non-linear behaviour. We used a variable reflecting Saturdays and linear B-splines, with interior knots at transition dates between EQ1 and EQ2, and between EQ2 and post-EQ (May 16^th^ and July 6^th^, respectively). Splines are more flexible around the knots and this allows to fully capture non-linear trends^22,23^. We assessed the model’s goodness-of-fit by visual inspection of its residuals, where a good fit means 95% of the deviance residuals are between −2 and 2 and no big outliers are present.

We used R software (version 3.5.0) to perform all analyses and considered α = 0.05.

### Ethical considerations

Personal identifiers were used solely in the data verification process, and removed immediately after. We submitted this research protocol to the Tribhuvan University – Institute of Medicine’s ethics committee. Since this study used secondary data from routinely generated information, the Tribhuvan University – Institute of Medicine’s ethics committee deemed that informed consent was not necessary, and they provided clearance to undertake this study (Ref. 381(6-11-E)^2^/074/075).

## Results

### Descriptive overview

**Figure 1:**
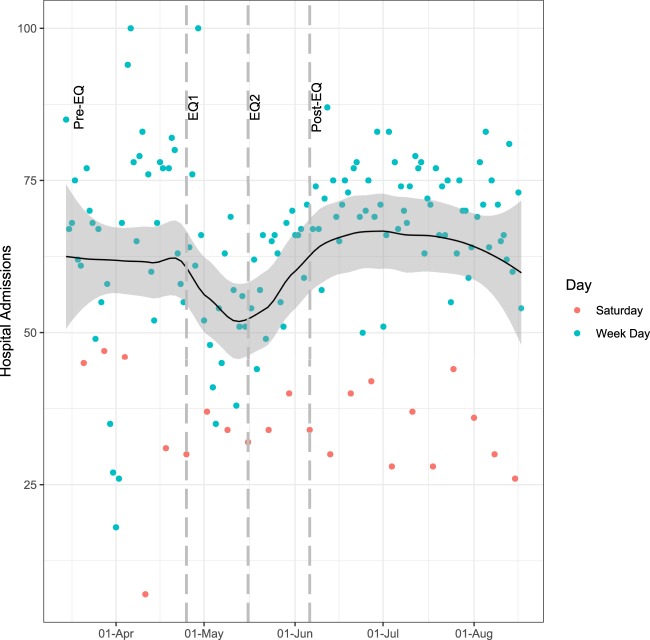
Evolution of number of admissions over time and smoothing trend. The smoothing curve is a local polynomial regression with span 0.5, where local regressions used to produce the curve incorporate 50% of the total nearest data points. The shaded area corresponds to the 95% confidence interval of the local polynomial regression. Red dots correspond to Saturdays, the weekly non-working day in Nepal. Dashed lines delimit different periods: pre-earthquake (pre-EQ), acute earthquake (EQ1), post-acute earthquake (EQ2), post-earthquake (post-EQ).

We included 9,596 admissions occurring between March 15^th^ and August 17^th^ 2015. Supplementary Figs. S1 and S2 show the number of observations included in each analysis and describe missing data. Although the number of daily admissions varied before the earthquake, they were lower in both EQ1 and EQ2, followed by a return to pre-EQ trends in the post-EQ period, as shown in Fig. 1.

Table 1 shows admissions by earthquake period, diagnostic category, sex, and age group. The post-EQ period contained 49% of all admissions, followed by the pre-EQ period with 26%. Overall, the most common causes of admission were injuries, pregnancy-related conditions, diseases of the digestive system, respiratory diseases, genitourinary diseases, and factors influencing health status and contact with health services. In our sample, this last category included a miscellaneous of conditions that i) arise when a person encounters health services for some specific purpose, such as to receive limited care or service for a current condition, to donate an organ or tissue, or to receive follow-up care; or that ii) influence the person’s health status but are not in themselves an illness or an injury, such as the outcome of delivery or a postsurgical state.

**Table 1:** Absolute and relative frequency of admissions by earthquake period, diagnostic category, sex, and age group.

		All periods, N (%)	pre-EQ, N (%)	EQ1, N (%)	EQ2, N (%)	post-EQ, N (%)
ICD-10 category	Perinatal conditions	136 (1.5)	44 (1.9)	13 (1.2)	6 (0.5)	73 (1.7)
	Infectious and parasitical diseases	352 (4.0)	89 (3.8)	22 (2.1)	46 (4.1)	195 (4.6)
	Congenital conditions	110 (1.2)	20 (0.9)	3 (0.3)	10 (0.9)	77 (1.8)
	Blood forming organ and immune system diseases	120 (1.4)	28 (1.2)	9 (0.9)	23 (2.0)	60 (1.4)
	Diseases of the circulatory system	408 (4.6)	99 (4.2)	50 (4.8)	54 (4.7)	205 (4.8)
	Diseases of the digestive system	954 (10.8)	266 (11.3)	58 (5.5)	136 (12.0)	494 (11.6)
	Diseases of the ear and mastoid process	128 (1.4)	38 (1.6)	8 (0.8)	18 (1.6)	64 (1.5)
	Genitourinary diseases	879 (10.0)	282 (11.9)	45 (4.3)	100 (8.9)	452 (10.6)
	Musculoskeletal and connective tissue diseases	109 (1.2)	15 (0.6)	5 (0.5)	12 (1.1)	77 (1.8)
	Neurological diseases	217 (2.5)	40 (1.7)	24 (2.3)	25 (2.2)	128 (3.0)
	Respiratory diseases	899 (10.2)	297 (12.6)	78 (7.4)	115 (10.2)	409 (9.6)
	Skin and subcutaneous diseases	123 (1.4)	47 (2.0)	5 (0.5)	17 (1.5)	54 (1.3)
	Endocrine, nutritional and metabolic diseases	256 (2.9)	62 (2.6)	18 (1.7)	36 (3.2)	140 (3.3)
	Contact with health services	902 (10.2)	210 (8.9)	125 (11.9)	119 (10.5)	448 (10.5)
	Injuries and other external causes	1284 (14.6)	263 (11.1)	405 (38.5)	160 (14.2)	456 (10.7)
	Mental and behavioural disorders	202 (2.3)	52 (2.2)	29 (2.8)	27 (2.4)	94 (2.2)
	Neoplasms	424 (4.8)	107 (4.5)	21 (2.0)	64 (5.7)	232 (5.4)
	Pregnancy, childbirth and the puerperium	1061 (12.0)	326 (13.8)	120 (11.4)	125 (11.1)	490 (11.5)
	Other not elsewhere classified	186 (2.1)	56 (2.4)	14 (1.3)	21 (1.9)	95 (2.2)
Age	0–4 years	770 (8.0)	206 (8.7)	64 (6.1)	81 (7.2)	360 (8.4)
	5–14 years	889 (9.3)	215 (9.1)	99 (9.4)	92 (8.1)	409 (9.6)
	15–49 years	5555 (57.9)	1353 (57.3)	633 (60.1)	651 (57.6)	2512 (58.8)
	≥ 50 years	2379 (24.8)	589 (24.9)	257 (24.4)	306 (27.1)	990 (23.2)
Sex	Male	4178 (43.6)	1001 (42.4)	443 (42.1)	502 (44.4)	1864 (43.6)
	Female	5416 (56.4)	1362 (57.6)	610 (57.9)	628 (55.6)	2407 (56.4)
Total		**9596**	**2537**	**1128**	**1205**	**4726**

ICD-10 category names are simplified from their original designations for readability. Sums do not always add up due to missing values. pre-EQ: pre-earthquake period; EQ1: acute earthquake period; EQ2: post-acute earthquake period; post-EQ: post-earthquake period; ICD-10: international classification of diseases, 10^th^ revision.

Women accounted for 56% of all admissions, while children and adolescents under 15 years of age represented 17% of all admissions.

### Length of hospital stay (LOS)

LOS distribution was right-skewed, ranging from 0 to 175 days, with an average of 7.7 days (sd 9.59), a median of 5 days, and an interquartile range of 3 to 9 days. Table 2 gives an overview of mean and median LOS in each study period. LOS was 19.7% longer during EQ1 than during the pre-EQ period (CI: 12.7–27.2; p < 0.001). There are no significant differences in LOS between any of the other earthquake periods.

**Table 2:** Central tendency and dispersion statistics for length of hospital stay. All values shown are in days. sd: standard deviation.

Period	Mean (sd)	Median	Range (minimum-maximum)	Interquartile range
Pre-EQ	7.05 (8.30)	5	0–110	3–8
EQ1	9.80 (12.46)	5	0–106	3–12
EQ2	7.49 (8.88)	5	0–87	3–9
Post-EQ	7.58 (9.58)	5	0–175	3–9

In comparison to pre-EQ, LOS for admissions due to injury and due to contact with health services were 57.3% (CI: 37.0–80.7; p < 0.001) and 21.0% (CI: 0.1–46.0; p = 0.046) longer during EQ1, respectively. In contrast, LOS for admissions related to respiratory diseases decreased by 21.6% in EQ1(CI: 7.1–34.6; p = 0.008).

### Association between EQ period and ICD-10 category

In the bivariate analyses, earthquake period was significantly associated with age groups and ICD-10 categories (chi-square test, p = 0.029 and <0.001, respectively). The share of small children (0–4years) was relatively low in EQ1, while the share of older people (50 years and older) relatively increased in EQ2 and post-EQ.

Table 3 presents the results of the logistic regressions, showing that the odds of admission due to injuries were significantly higher in EQ1 and EQ2, compared to pre-EQ. The odds for admissions due to contact with health services were higher in EQ1, EQ2, and post-EQ. Pregnancy-related conditions and respiratory diseases had lower odds of admission in all study periods compared to pre-EQ. The odds of admission during EQ1 were significantly lower for infectious diseases, diseases of digestive system, diseases of ear and mastoid, skin diseases, and neoplasms, compared to pre-EQ. Genitourinary diseases had lower odds of admission during both EQ1 and EQ2. Congenital, neurological, and musculoskeletal diseases had increased odds of admission only in post-EQ. The most frequent conditions seen at TUTH for congenital diseases included congenital hydrocephalus, malformations of face and neck, and malformation of male organs. The most common neurological conditions included hemiplegia, para or tetraplegia, and other paralytic conditions. Musculoskeletal disorder-related admissions included mostly joint disorders and autoimmune diseases.

**Table 3:** Measures of association of ICD-10 Category.

ICD-10 Category		aOR (95% CI)	se	p-value	aOR (95% CI)	se	p-value	aOR (95% CI)	se	p-value
Perinatal conditions	Ref	0.66 (0.35–1.23)	0,21	0.190	0.28 (0.12–0.66)	0,12	0.004**	0.91 (0.63–1.33)	0,18	0.642
Infectious and parasitical diseases	Ref	0.56 (0.35–0.89)	0,13	0.015*	1.09 (0.76–1.57)	0,2	0.643	1.22 (0.94–1.57)	0,16	0.135
Congenital conditions	Ref	0.36 (0.11–1.22)	0,22	0.099	1.11 (0.52–2.40)	0,44	0.781	2.16 (1.31–3.55)	0,55	0.002**
Blood forming organ and immune system diseases	Ref	0.71 (0.33–1.52)	0,27	0.379	1.74 (1.00–3.03)	0,49	0.051	1.19 (0.76–1.87)	0,27	0.456
Disease of the circulatory system	Ref	1.16 (0.81–1.65)	0,21	0.411	1.09 (0.78–1.54)	0,19	0.610	1.18 (0.92–1.52)	0,15	0.184
Disease of the digestive system	Ref	0.45 (0.34–0.61)	0,07	<0.001***	1.05 (0.84–1.31)	0,12	0.688	1.03 (0.88–1.21)	0,08	0.737
Diseases of the ear and mastoid process	Ref	0.46 (0.21–0.98)	0,18	0.045*	1.05 (0.59–1.85)	0,31	0.880	0.90 (0.60–1.35)	0,19	0.607
Genitourinary diseases	Ref	0.32 (0.24–0.45)	0,05	<0.001***	0.70 (0.55–0.90)	0,09	<0.001***	0.87 (0.74–1.02)	0,07	0.085
Musculoskeletal and connective tissue diseases	Ref	0.74 (0.27–2.04)	0,38	0.559	1.68 (0.78–3.61)	0,66	0.183	2.84 (1.63–4.94)	0,81	<0.001***
Neurological diseases	Ref	1.40 (0.84–2.33)	0,36	0.200	1.32 (0.80–2.20)	0,34	0.277	1.80 (2.26–2.58)	0,33	0.001**
Respiratory diseases	Ref	0.57 (0.43–0.74)	0,07	<0.001***	0.77 (0.61–0.97)	0,09	0.029*	0.73 (0.62–0.86)	0,06	<0.001***
Skin and subcutaneous diseases	Ref	0.23 (0.09–0.59)	0,11	0.002**	0.75 (0.43–1.31)	0,21	0.313	0.62 (0.42–0.92)	0,13	0.019*
Endocrine, nutritional and metabolic diseases	Ref	0.64 (0.38–1.10)	0,17	0.107	1.18 (0.78–1.80)	0,25	0.438	1.31 (0.96–1.78)	0,2	0.084
Contact with Health Services	Ref	1.47 (1.14–1.89)	0,19	0.003**	1.31 (1.02–1.69)	0,17	0.038*	1.23 (1.03–1.48)	0,11	0.024*
Injuries and other external causes	Ref	5.33 (4.44–6.40)	0,5	<0.001***	1.32 (1.07–1.64)	0,14	0.011*	0.94 (0.80–1.11)	0,08	0.456
Mental and behavioural Disorders	Ref	1.21 (0.76–1.92)	0,29	0.418	1.05 (0.65–1.68)	0,25	0.852	0.97 (0.69–1.37)	0,17	0.860
Neoplasms	Ref	0.43 (0.27–0.69)	0,1	<0.001***	1.22 (0.88–1.69)	0,2	0.225	1.26 (0.99–1.59)	0,15	0.060
Pregnancy, childbirth and the puerperium	Ref	0.74 (0.59–0.94)	0,09	0.012*	0.75 (0.60–0.95)	0,09	0.016*	0.77 (0.65–0.90)	0,06	<0.001***
Other not elsewhere classified	Ref	0.59 (0.33–1.06)	0,18	0.080	0.81 (0.49–1.34)	0,21	0.410	0.94 (0.67–1.32)	0,16	0.716

*p < 0.05; **p < 0.01; ***p < 0.001. aOR: adjusted odds ratio; CI: Confidence Interval; se: standard error; Ref: Reference category.

Each ICD-10 category is compared to all other categories combined. Only ICD-10 categories with more than 70 observations are included. We removed the variable age group for diagnostic category “perinatal conditions” (concerning only children aged 0–4 years), and the sex variable for pregnancy-related conditions (concerning only females). The number of observations included was n = 8817. The ICD-10 category names presented are simplified from the original denomination for readability. Also for readability concerns we are not showing aOR for sex and age groups, although these variables have been adjusted for. A full table can be consulted in Supplementary Table S3.

We checked for interaction effects of earthquake period with age group and with sex, but these models were not significantly better, so we discarded them.

### Impact on hospital admissions

**Figure 2:**
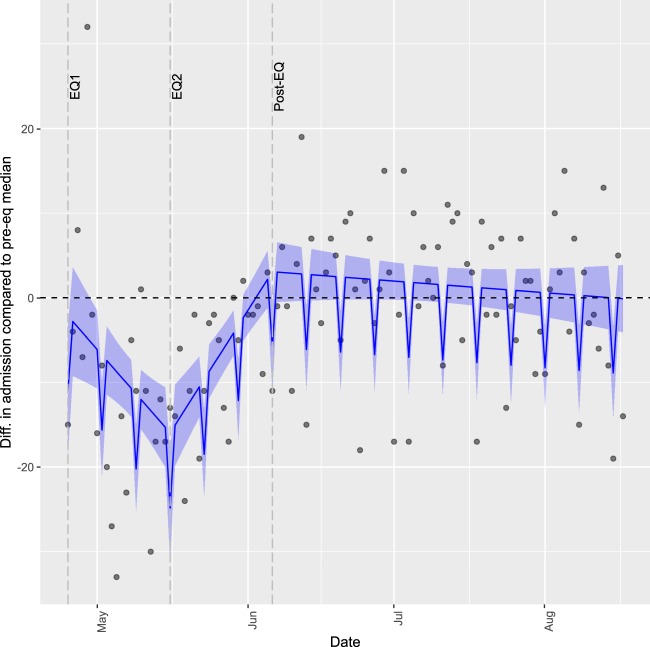
Difference of daily hospital admissions, compared to baseline pre-EQ median. The line and shade represent the predicted values resulting from our model and their 95% confidence interval. Diff. = Difference; pre-eq= pre-earthquake period from March 15^th^ to April 24^th^ 2015.

In the pre-EQ period, the median number of daily admissions was 68 on weekdays and 45 on Saturdays, which we considered the baseline for further analyses. As shown in Fig. 2, the difference of hospital admissions was negative in both EQ1 and EQ2, meaning there were less admissions in these periods than in the pre-EQ baseline. While in EQ1, the number of daily admissions decreased, it had an ascending pattern in EQ2. In post-EQ, the difference of admissions had a tendency towards zero.

**Figure 3:**
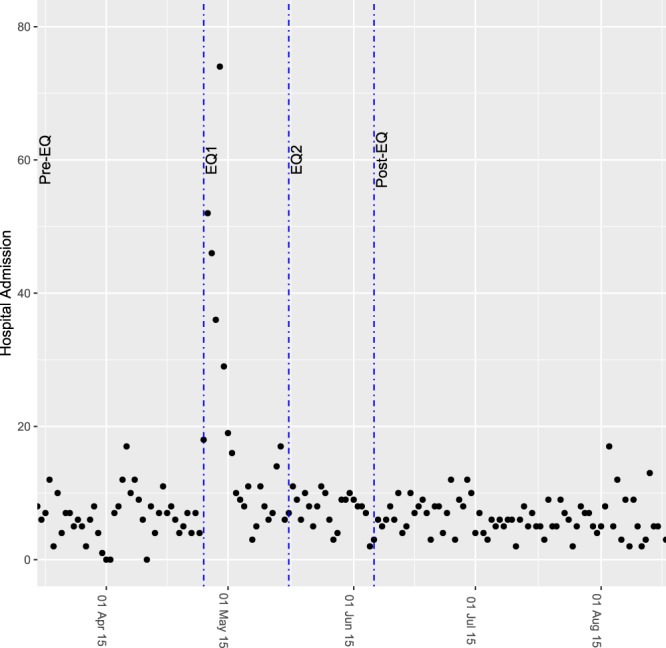
Daily admissions due to injury in all periods. Dashed lines differentiate earthquake periods. pre-EQ: pre-earthquake period (from March 15^th^ to April 24^th^); EQ1: acute earthquake period (from April 25^th^ to May 15^th^); EQ2: post-acute earthquake period (from May 16^th^ to June 5^th^); post-EQ: post-earthquake period (from June 6^th^ to August 17^th^).

The model residuals do not show a particular remaining pattern although a big outlier is present (Supplementary Fig. S4), corresponding to the fifth day after the earthquake, where admissions were particularly high due to a peak of injury admissions, as shown in Fig. 3. According to this model, the cumulative losses in admissions at the end of EQ1 reached 210 hospital admissions (95% CI: 112–307), whereas at the end of EQ2 they totalled 381 (95% CI: 206–556).

## Discussion

Our results show that, in the aftermath of the 2015 earthquake, the pattern of hospital admissions varied with time. In the three weeks following the earthquake (EQ1), length of stay (LOS) for admissions was significantly longer than in other periods. This was mostly due to injury admissions, in which LOS highly increased. There were higher odds of admission due to injuries and contact with health services, compared with the pre-EQ period, and relative to other admissions. There was also a significant relative decrease of admissions due to infectious diseases, neoplasms, pregnancy-related conditions, ear and mastoid diseases, digestive diseases, respiratory diseases, skin diseases, and genitourinary diseases. Finally, the overall number of admissions decreased significantly in EQ1 in comparison to the pre-EQ scenario, remaining significantly low in EQ2, and returning to pre-EQ trends in post-EQ.

The fact that injury admissions occurring in EQ1 were particularly long may relate to earthquake injury characteristics linked with severity and frequent complications^24^, or because many severe cases were probably referred to TUTH since it is a reference hospital, and it was functional after the earthquake^15^. In addition, external factors may have increased LOS: a disrupted transport network, or destroyed housing, may have delayed patient discharge until an improvement of the situation. Nevertheless, LOS for respiratory diseases substantially decreased in EQ1. This is probably because such admissions were shortened to ensure all earthquake victims in need were admitted and appropriately treated. These findings are in line with a study in a rural hospital in Nepal describing the patient load in the three weeks after the earthquake, which found that earthquake-related conditions had significantly longer hospitalizations compared to other non-earthquake related admissions^17^.

There was a relative increase of admissions due to injuries and due to other factors influencing contact with health services in EQ1; while admissions due to other ICD-10 categories substantially decreased. As explained in a conceptual model suggested by von Schreeb *et al*., the need for hospital care due to earthquake injuries is concentrated in the days after the earthquake, while other elective and less urgent conditions are deferred^2^. Shortly after, there is a need for hospital care for trauma-related complications, which could explain why admissions due to factors influencing health status and contact with health services are high in EQ1 and EQ2. A previous study identified this diagnostic category in some of the earthquake victims who were admitted in TUTH^15^. The high probability of admissions in this category in post-EQ may be due to an accumulation of interventions that re-started after weeks of being interrupted, such as the donation of organs and tissues by healthy donors. Admissions due to congenital diseases, musculoskeletal diseases, and neurological diseases increased in post-EQ, which could also correspond to an accumulation of elective care interrupted in EQ1 and EQ2. An unexpected finding was the sustained decrease of respiratory conditions in post-EQ in comparison to pre-EQ, as the literature reports medium-term increases of respiratory diseases after earthquakes^4,5^. The fact that our full study period corresponds to warmer months in Nepal, and that the population is relatively young, could be an explanation for this observation. Severe respiratory diseases requiring in-hospital treatment are less often expected, also because people were not particularly exposed to water or humidity during this earthquake – as opposed to hurricanes or earthquakes followed by tsunamis, characterized by an increase of pneumonia cases^3,4,25^. As such, most respiratory consequences of the 2015 earthquake would be reflected in outpatient care. A study in Patan Hospital, in the vicinity of Kathmandu, showed that there were significantly more emergency visits due to cardiovascular, psychiatric, respiratory, and hematologic conditions in the four months after the earthquake, compared to the same period one year before^19^.

Our findings show that pregnancy-related admissions decreased immediately after the earthquake, and remained low in the long-term. A study following typhoon Haiyan also identified an immediate decrease of pregnancy-related admissions, suggesting that lack of access may have caused an increase in unsafe deliveries with lack of adequate care^3^. An ethnographic study in rural Nepal showed that, after the earthquake, several women preferred delivering at home rather than at a health facility, making it more difficult to refer to a hospital when needed^26^. The transfer from basic delivery facilities to higher levels of care was disrupted after the earthquake due to road destruction^26^, which could further explain a sustained decrease in pregnancy-related admissions at TUTH. There are reports of operational mobile reproductive health clinics set up by the United Nations Population Fund (UNFPA) and the Ministry of Health in the initial response phase to address specific maternal care needs^26^, but there is very little information describing long term changes in maternal health. Some admissions related to the outcome of delivery, however, are categorized under “factors influencing health status and contact with health services”, which merits a more detailed examination combined with a more in-depth analysis of the category Pregnancy, Childbirth, and the Puerperium. In addition, more comprehensive studies can shed light on the external context’s influence on maternal healthcare. Regardless of this, it is essential to ensure appropriate care for pregnant women in the months following a disaster.

It is known that earthquakes cause a sharp increase of medical needs due to injuries, many of them requiring surgical and in-hospital treatment^2,27^. Yet, our final analysis showed that, despite an initial peak of admissions on the fifth day after the earthquake, the total number of admissions dropped in the weeks after the earthquake, and slowly increased back to baseline levels after six weeks. During this six-week period, our model estimates that there were 381 fewer admissions than if the pre-EQ trend had continued. TUTH was functioning immediately after the earthquake, with victims and visitors arriving within 20 minutes after the shake. The emergency department put in place its mass casualty triage system, and increased its space by using other buildings as specific triage color areas. It is possible the emergency department treated many of the victims, who in normal circumstances would have been admitted, in an outpatient basis. Despite the lack of documentation to support this, it is likely that only the most severe cases were admitted in the hospital, which themselves were very resource-consuming, suspending all other non-urgent admissions. This is supported by the fact that admissions occurring during EQ1 had higher LOS, but would benefit from more research exploring other contextual factors.

Finally, there is no clear compensating bump for the total number of admissions in the beginning of the post-EQ period, but an accumulation of admissions of specific diagnostic categories may have happened, as indicated in the logistic regression, for congenital, musculoskeletal, and neurological diseases. This suggests an attempt to resume delayed work, as von Schreeb *et al*.’s conceptual model proposes^2^.

Our study had some limitations, including that it used a large dataset from routine hospital activities, not created for research purposes, with a limited number of variables. There may have been a categorization bias in the diagnosis classification, since it often influences financial compensation. However, by using broad ICD-10 categories, we reduced this bias. Data from the earthquake periods may be less complete because of the hectic situation at the time, and it is possible that not all information was systematically registered. Since we completed and verified most of our findings with an additional earthquake victim dataset, which we verified with original patient files, we believe this is a minor issue. Our study compared the post-disaster situation with a relatively short baseline period, and did not take seasonality into account. Seasonality could partially have been overcome by using annual trends from previous years, but this information was not available. Nevertheless, in the post-EQ period, the number of admissions returned to baseline, suggesting a fairly low seasonal variation of daily hospital admissions at TUTH in our study period. When we computed the baseline number of daily admissions, we differentiated for the only non-working day in Nepal. However, we could not capture variations related to festive holidays, strikes, or other events that may affect admission patterns.

## Conclusions

Earthquakes heavily disrupt the functioning of tertiary hospitals. Our study provides useful information for tertiary hospitals in seismic and low-resource settings. Because of the increase of injured patients with long admissions, hospitals should be prepared to quickly mobilize resources to treat high numbers of injuries in the event of an earthquake. But the overall number of admissions decreased in the weeks following the earthquake, mostly due to a strong decrease of admissions due to other causes, which were also shorter than usual immediately after the earthquake. To prevent negative health outcomes from conditions other than injuries, efforts are needed to ensure accessibility to the hospital and a quick return to normality. The relative decrease of pregnancy-related conditions is concerning because deliveries are expected to remain stable despite the earthquake, suggesting deliveries are taking place elsewhere or without the presence of a skilled attendant. While more research is needed to understand earthquake impact on maternal healthcare at the hospital level and beyond, a priority is to ensure community capacity to manage urgent maternal health needs in remote areas after earthquakes.

## Supplementary information

Supplementary files.

## Data Availability

The dataset that supports the findings of this study is available from the Harvard dataverse repository, 10.7910/DVN/OQ3W5K.
