# Supplementary material for: Changes in patient admissions after the 2015 Earthquake: a tertiary hospital-based study in Kathmandu, Nepal
Source: Sci Rep. 2020 Mar 18;10:4956. doi: 10.1038/s41598-020-61901-7 (PMC7080816; doi:10.1038/s41598-020-61901-7)
Supplement: Supplementary file 1 — Supplementary files. [file 41598_2020_61901_MOESM1_ESM.pdf]

Changes in patient admissions after the 2015 Earthquake: a tertiary hospital-based study in Kathmandu, Nepal

Maria Moitinho de Almeida[1*]; Benjamin-Samuel Schlüter[1]; Joris Adriaan Frank van Loenhout[1]; Sunil Singh Thapa[2]; K.C. Kumar [3]; Ravikant Singh[4]; Debarati Guha-Sapir[1]; Deepak Prakash Mahara[2]

[1]Centre for Research on the Epidemiology of Disasters, Institute of Health and Society, University of Louvain. 30, clos chapelle-aux-champs, 1200 Brussels, Belgium

[2]Department of Orthopedics, Tribhuvan University Teaching Hospital. Maharajgunj Rd, Kathmandu 44600, Nepal

[3]Tribhuvan University Teaching Hospital. Maharajgunj Rd, Kathmandu 44600, Nepal

[4]Doctors for You India, Natwar Parikh Compound, Near India Oil Nagar, Govandi. Mumbai 400043, Maharashtra, India

* corresponding author:

Maria Moitinho de Almeida; maria.rodrigues@uclouvain.be; +32(0)27643368

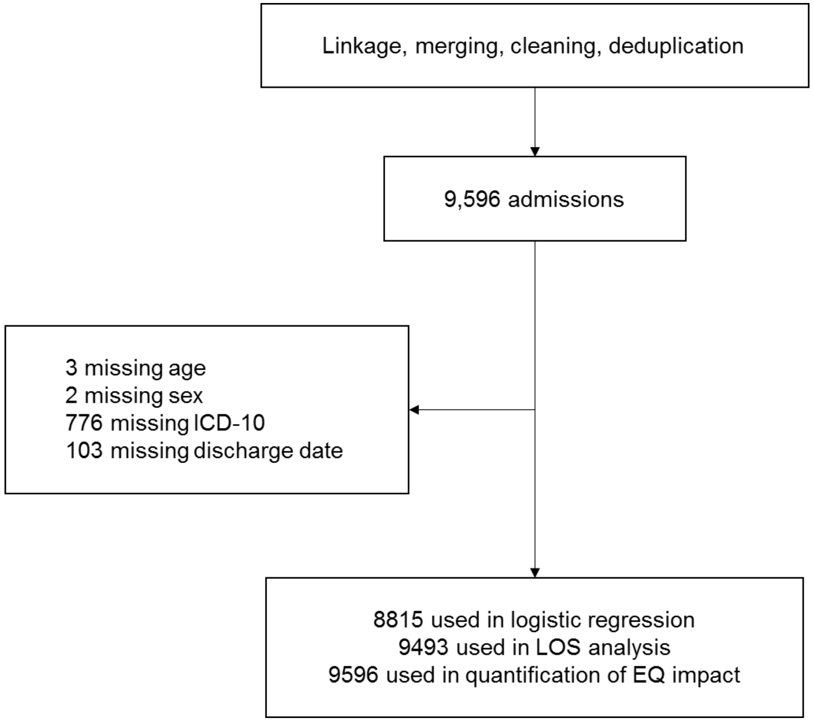

Supplementary file S1: Flowchart of total observations, missing values, and observations used in the analyses
ICD-10: International Classification of Diseases, 10th edition. LOS: Length of hospital Stay. EQ: Earthquake.

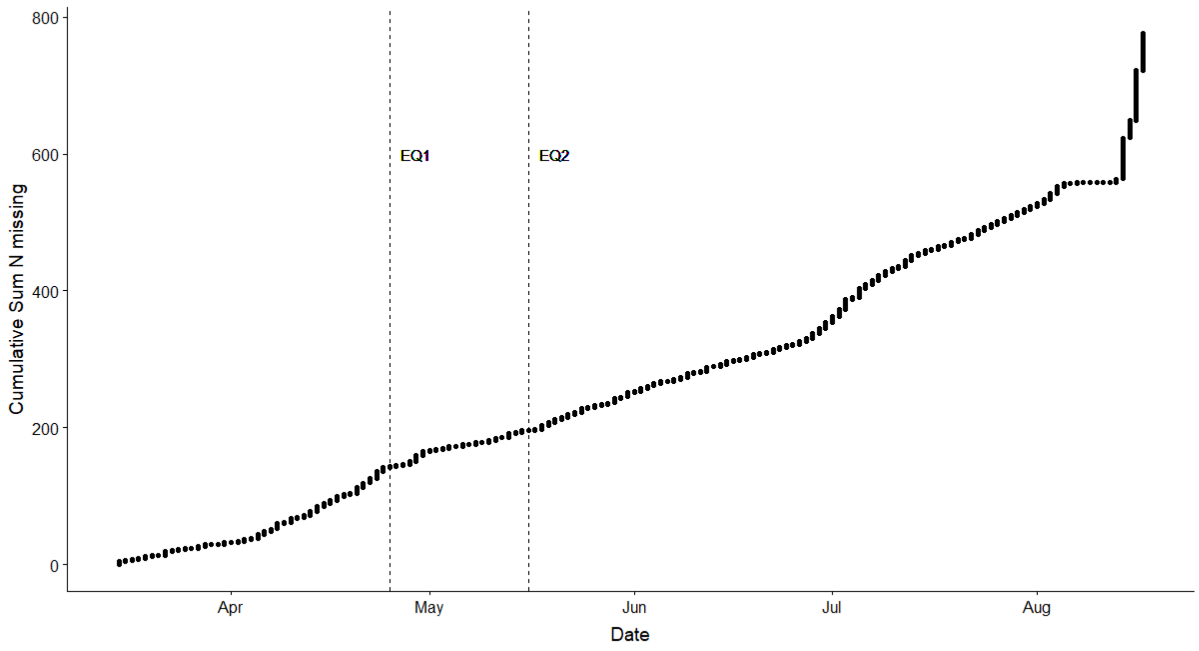

Supplementary file S2: Cumulative sum of missing ICD-10 values over time

		Earthquake Period						Age group (years)							Sex		
**ICD-10 Category**	Pre EQ	EQ 1		EQ 2		Post EQ		15-49	<5		5-14		>=50		Male	Female	
		aOR (95% CI)	P-value	aOR (95% CI)	P-value	aOR (95% CI)	P-value		aOR (95% CI)	P-value	aOR (95% CI)	P-value	aOR (95% CI)	P-value		aOR (95% CI)	P-value
Perinatal conditions	Ref	0·66 (0·35-1·23)	0·190	0·28 (0·12-0·66)	0·004**	0·91 (0·63-1·33)	0·642	N/A	N/A		N/A		N/A		Ref	0·85 (0·61-1·19)	0·349
Infectious and parasitical diseases	Ref	0·56 (0·35-0·89)	0·015*	1·09 (0·76-1·57)	0·643	1·22 (0·94-1·57)	0·135	Ref	1·75 (1·25-2·47)	0·001**	1·61 (1·16-2·24)	0·005**	1·07 (0·82-1·40)	0·627	Ref	0·58 (0·47-0·73)	<0·001***
Congenital conditions	Ref	0·36 (0·11-1·22)	0·099	1·11 (0·52-2·40)	0·781	2·16 (1·31-3·55)	0·002**	Ref	4·55 (2·83-7·31)	<0·001***	2·69 (1·58-4·56)	<0·001***	0·58 (0·30-1·11)	0·099	Ref	0·62 (0·42-0·92)	0·017*
Blood forming organ and immune system diseases	Ref	0·71 (0·33-1·52)	0·379	1·74 (1·00-3·03)	0·051	1·19 (0·76-1·87)	0·456	Ref	0·86 (0·39-1·90)	0·710	2·20 (1·31-3·71)	0·003**	1·26 (0·81-1·96)	0·302	Ref	1·27 (0·87-1·85)	0·220
Disease of the circulatory system	Ref	1·16 (0·81-1·65)	0·411	1·09 (0·78-1·54)	0·610	1·18 (0·92-1·52)	0·184	Ref	1·00 (0·63-1·60)	0·996	0·80 (0·50-1·30)	0·371	3·86 (3·10-4·82)	<0·001***	Ref	0·75 (0·61-0·92)	0·006**
Disease of the digestive system	Ref	0·45 (0·34-0·61)	<0·001***	1·05 (0·84-1·31)	0·688	1·03 (0·88-1·21)	0·737	Ref	0·66 (0·49-0·89)	<0·001***	1·07 (0·84-1·35)	0·605	1·53 (1·31-1·79)	<0·001***	Ref	0·59 (0·51-0·67)	<0·001***
Diseases of the ear and mastoid process	Ref	0·46 (0·21-0·98)	0·045*	1·05 (0·59-1·85)	0·880	0·90 (0·60-1·35)	0·607	Ref	0·43 (0·16-1·19)	0·104	5·67 (3·86-8·32)	<0·001***	0·22 (0·09-0·50)	<0·001***	Ref	0·72 (0·50-1·04)	0·080
Genitourinary diseases	Ref	0·32 (0·24-0·45)	<0·001***	0·70 (0·55-0·90)	<0·001***	0·87 (0·74-1·02)	0·085	Ref	0·56 (0·41-0·77)	<0·001***	0·82 (0·63-1·06)	0·127	1·06 (0·90-1·25)	0·487	Ref	0·85 (0·74-0·98)	0·030*
Musculoskeletal and connective tissue diseases	Ref	0·74 (0·27-2·04)	0·559	1·68 (0·78-3·61)	0·183	2·84 (1·63-4·94)	<0·001***	Ref	0·53 (0·21-1·34)	0·183	2·04 (1·22-3·39)	0·006**	0·85 (0·52-1·39)	0·512	Ref	0·60 (0·40-0·89)	0·011*

Neurological diseases	Ref	1·40 (0·84-2·33)	0·200	1·32 (0·80-2·20)	0·277	1·80 (2·26-2·58)	0·001**	Ref	2·19 (1·44-3·33)	<0·001***	1·77 (1·16-2·70)	<0·001***	1·38 (0·98-1·92)	0·061	Ref	0·69 (0·52-0·91)	0·009**
Respiratory diseases	Ref	0·57 (0·43-0·74)	<0·001***	0·77 (0·61-0·97)	0·029*	0·73 (0·62-0·86)	<0·001***	Ref	1·85 (1·46-2·33)	<0·001***	1·41 (1·11-1·79)	0·004**	1·68 (1·42-1·98)	<0·001***	Ref	0·54 (0·46-0·62)	<0·001***
Skin and subcutaneous diseases	Ref	0·23 (0·09-0·59)	0·002**	0·75 (0·43-1·31)	0·313	0·62 (0·42-0·92)	0·019*	Ref	0·81 (0·38-1·70)	0·572	2·04 (1·24-3·36)	0·005**	1·07 (0·69-1·67)	0·766	Ref	0·57 (0·39-0·83)	0·004**
Endocrine, nutritional and metabolic diseases	Ref	0·64 (0·38-1·10)	0·107	1·18 (0·78-1·80)	0·438	1·31 (0·96-1·78)	0·084	Ref	0·88 (0·47-1·66)	0·698	0·78 (0·41-1·46)	0·435	4·02 (3·06-5·29)	<0·001***	Ref	1·21 (0·93-1·56)	0·156
Contact with Health Services	Ref	1·47 (1·14-1·89)	0·003**	1·31 (1·02-1·69)	0·038*	1·23 (1·03-1·48)	0·024*	Ref	0·16 (0·10-0·27)	<0·001***	0·08 (0·04-0·16)	<0·001***	0·07 (0·04-0·10)	<0·001***	Ref	12·42 (9·21-16·76)	<0·001***
Injuries and other external causes	Ref	5·33 (4·44-6·40)	<0·001***	1·32 (1·07-1·64)	0·011*	0·94 (0·80-1·11)	0·456	Ref	1·04 (0·82-1·32)	0·720	2·08 (1·72-2·51)	<0·001***	1·02 (0·87-1·19)	0·824	Ref	0·47 (0·41-0·53)	<0·001***
Mental and behavioural Disorders	Ref	1·21 (0·76-1·92)	0·418	1·05 (0·65-1·68)	0·852	0·97 (0·69-1·37)	0·860	Ref	0·39 (0·21-0·76)	0·005**	0·45 (0·26-0·79)	0·005**	0·49 (0·34-0·71)	<0·001***	Ref	0·32 (0·24-0·43)	<0·001***
Neoplasms	Ref	0·43 (0·27-0·69)	<0·001***	1·22 (0·88-1·69)	0·225	1·26 (0·99-1·59)	0·060	Ref	1·35 (0·90-2·04)	0·146	0·44 (0·24-0·81)	0·009**	3·94 (3·17-4·89)	<0·001***	Ref	0·96 (0·79-1·18)	0·711
Pregnancy, childbirth and the puerperium	Ref	0·74 (0·59-0·94)	0·012*	0·75 (0·60-0·95)	0·016*	0·77 (0·65-0·90)	<0·001***	Ref	0·07 (0·04-0·13)	<0·001***	0·01 (0·01-0·03)	<0·001***	0·01 (0·01-0·03)	<0·001***	N/A	N/A	
Other not elsewhere classified	Ref	0·59 (0·33-1·06)	0·080	0·81 (0·49-1·34)	0·410	0.94 (0.67-1.32)	0·716	Ref	4·53 (3·03-6·78)	<0·001***	3·19 (2·08-4·89)	<0·001***	1·54 (1·04-2·28)	0·031*	Ref	0·75 (0·55-1·02)	0·062

Supplementary Table S3: Results of completed logistic regression analyses
*p<0·05; **p<0·01; ***p<0·001. aOR: adjusted odds ratio; CI: Confidence Interval; Ref: Reference category.
Each ICD-10 category is compared to all other categories combined. Only ICD-10 categories with more than 70 observations are included. We removed the variable age group for diagnostic category “perinatal conditions”, as it only included children aged 0-4 years, and the sex variable for pregnancy-related conditions, as it only consisted of females. The number of observations included was n=8817. The ICD-10 category names presented are simplified from the original denomination for readability.

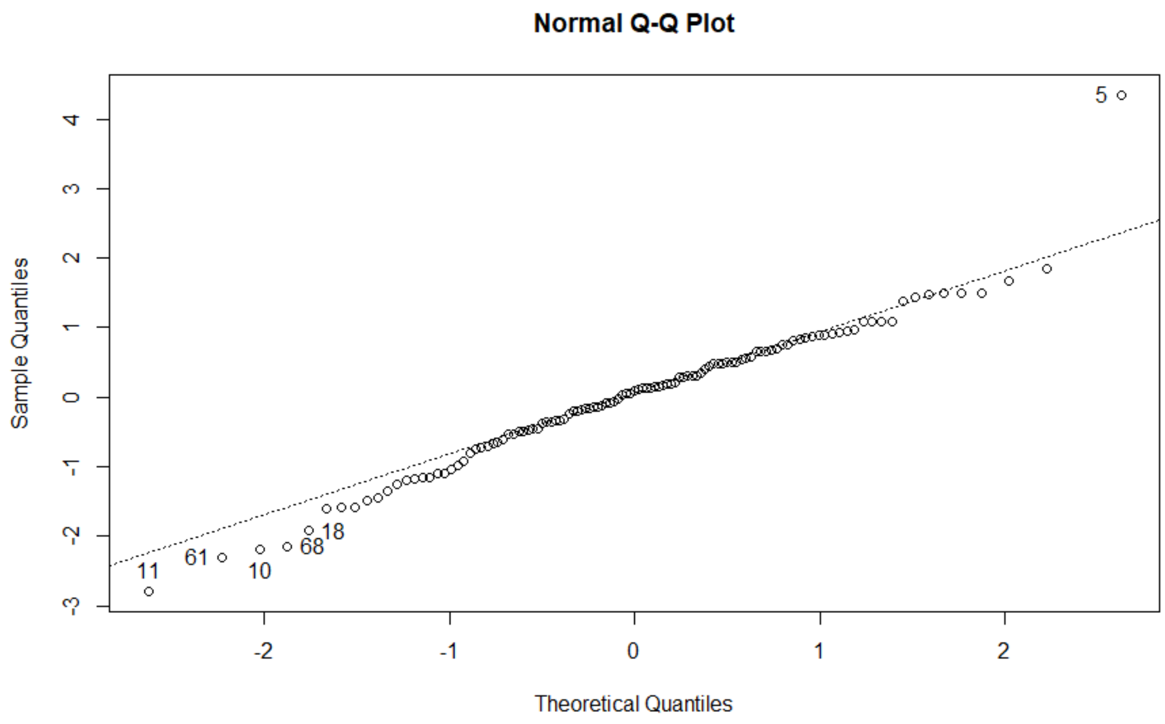

Supplementary File S4: Residuals distribution from the proposed model of differences of hospital admissions in EQ1, EQ2 and post-EQ with the median of admissions in the pre-EQ period.

We used Generalized Additive Models to model these differences and capture non-linear behavior. The baseline of comparison was the median of admissions on week days and Saturdays in the pre-EQ period.

A big outlier is present on the fifth day after the Earthquake that occurred on April 25th 2015. This correspondents to the highest peak of injury admissions.
